# Molecular Characterization of *Clostridium botulinum* Isolates from Foodborne Outbreaks in Thailand, 2010

**DOI:** 10.1371/journal.pone.0077792

**Published:** 2014-01-27

**Authors:** Piyada Wangroongsarb, Tomoko Kohda, Chutima Jittaprasartsin, Karun Suthivarakom, Thanitchi Kamthalang, Kaoru Umeda, Pathom Sawanpanyalert, Shunji Kozaki, Kazuyoshi Ikuta

**Affiliations:** 1 Department of Medical Sciences, National Institute of Health, Nonthaburi, Thailand; 2 Department of Veterinary Science, Osaka Prefecture University, Osaka, Japan; 3 Department of Microbiology, Osaka City Institute of Public Health and Environmental Sciences, Osaka, Japan; 4 Department of Medical Sciences, Nonthaburi, Thailand; 5 Department of Virology, Osaka University, Osaka, Japan; United States Army Medical Research Institute of Infectious Diseases, United States of America

## Abstract

**Background:**

Thailand has had several foodborne outbreaks of botulism, one of the biggest being in 2006 when laboratory investigations identified the etiologic agent as *Clostridium botulinum* type A. Identification of the etiologic agent from outbreak samples is laborious using conventional microbiological methods and the neurotoxin mouse bioassay. Advances in molecular techniques have added enormous information regarding the etiology of outbreaks and characterization of isolates. We applied these methods in three outbreaks of botulism in Thailand in 2010.

**Methodology/Principal Findings:**

A total of 19 cases were involved (seven each in Lampang and Saraburi and five in Maehongson provinces). The first outbreak in Lampang province in April 2010 was associated with *C. botulinum* type F, which was detected by conventional methods. Outbreaks in Saraburi and Maehongson provinces occurred in May and December were due to *C. botulinum* type A1(B) and B that were identified by conventional methods and molecular techniques, respectively. The result of phylogenetic sequence analysis showed that *C. botulinum* type A1(B) strain Saraburi 2010 was close to strain Iwate 2007. Molecular analysis of the third outbreak in Maehongson province showed *C. botulinum* type B8, which was different from B1–B7 subtype. The nontoxic component genes of strain Maehongson 2010 revealed that *ha*33, *ha*17 and *botR* genes were close to strain Okra (B1) while *ha*70 and *ntnh* genes were close to strain 111 (B2).

**Conclusion/Significance:**

This study demonstrates the utility of molecular genotyping of *C. botulinum* and how it contributes to our understanding the epidemiology and variation of *boNT* gene. Thus, the recent botulism outbreaks in Thailand were induced by various *C. botulinum* types.

## Introduction

In the past, Thailand has experienced several foodborne outbreaks of botulism. During December 1997, six people from the Maesot district in Tak province found ill after consumption of homemade canned bamboo shoots. In April 1998, an outbreak in Thawangpha district, Nan province affected nine people who were hospitalized, four of whom required mechanical ventilation [1], [2]. The biggest botulism outbreak occurred in Banluang district, Nan province, in March 2006, affecting 209 people who attended a local Buddhist festival, and ate bamboo shoots [3], [4]. Administration of antitoxin resulted in no causalities in these outbreaks.

Foodborne botulism is first diagnosed on the basis of the patient’s symptoms and food history. Isolation of *C. botulinum* from stool or gastric samples along with signs and symptoms are definitive indication of botulism. But recovery of the organism from food that does not contain demonstrable toxin is inconclusive. The diagnosis has been ascertained by detecting toxin in the patients’ serum, stool or in the food consumed before the onset of illness [5]. The mouse bioassay is the most widely accepted method for detecting botulinum neurotoxins (BoNTs) in serum and suspected foods. This assay has the desired sensitivity (< five mouse lethal units/mL), but it is cumbersome, time consuming (one to four days) and involves the use of large numbers of animal [6], [7]. Alternatively *in vitro* methods such as enzyme-linked immunosorbent assay (ELISA) [7]–[9] and an assay with a large immune-sorbent surface area (ALISSA) [10] require only 5–6 hrs for the detection and are as sensitive as the mouse bioassay [7]–[9]. They are rapid and have a wider detection range including foods, environmental and sera samples [7]–[10]. The evolving epidemics of foodborne diseases must be monitored and understood to implement appropriate prevention methods. Traditionally, characterization and identification of the botulism outbreak require recovery of the organism by conventional culture methodology [11], [12] and mouse bioassay for detection neurotoxin production [6], [13]. Rapid test kits, based on phenotypic characteristics of anaerobic bacteria have been developed, but their sensitivity and specificity remain inadequate for *C. botulinum* identification [14], [15]. In addition, reliable typing of strains can only made when the BoNT belongs to a known serotype. Recently, molecular techniques for detecting *boNT* gene have been introduced in an attempt to replace the consuming time conventional methods [16]–[18]. There are seven antigenic serotypes of BoNT (A through G). Serotypes A, B, E and rarely F, can affect humans, while serotypes C and D cause botulism in animals worldwide [19]. Type G has been isolated from soil and from cases of unexplained deaths in Switzerland [20], [21]. Types A and B are generally associated with outbreaks in temperate and warmer zones, with one type often predominating over the other. Proteolytic types A and B are linked to the majority of the outbreaks in the United States, China, South America and southern European countries, and the most frequently implicated foods are vegetables. In Central Europe the vehicle is most often a meat product with the causative organism being non-proteolytic type B strains. More than 90% of the outbreaks are caused by home-prepared/home-preserved foods [5]. Type E has a limited geographical distribution and occurs primarily in northern countries such as Canada, Finland, Japan, Norway, Sweden, Russia, and Alaska in the United States [22].

Molecular characterization of BoNTs relies on their sequences of the structural genes/proteins of *C. botulinum* and sequence variations within BoNTs of serotype have led to the designation of subtypes. BoNT type A (prominent due to its high toxicity and long duration of action) that is divided into five subtypes, A1–A5 [23]; type B has seven subtypes, B1–B7 [24], [25]; type E is classified into nine subtypes, E1–E9 [22], [26]; and type F is divided into F1–F7 subtypes [27]. There are no reported subtypes for C, D and G [28]. However, the BoNT C/D and D/C mosaic types are considered to be BoNT C and D subtypes, respectively [29]–[31].

In 2010, three distinct outbreaks were reported, two in April and May in Lampang and Saraburi provinces, respectively each with seven cases, and a third outbreak in December 2010 in Maehongson province with five cases. This paper describes the molecular characterization of the *C. botulinum* isolates from these three outbreaks in Thailand.

## Materials and Methods

For the first outbreak in Lampang province, four stool samples, six serum samples and five food items (wild boar meat, sour meat samples and three others suspected food samples) were collected during 20 April to 17 May 2010. During the second outbreak in Saraburi province, four stool samples, three sera samples and four food items (the plastic wrap from pork sausages sample, pickled vegetables and the cans that these samples were retained and sour pork) were obtained from 17–25 May, 2010. Suspected samples in the third outbreak in Maehongson province were two stool samples, four sera samples and two fermented soy bean samples during 16–18 December, 2010. All samples were sent to the Anaerobic Bacteria Section, National Institute of Health, Thailand for identification. One gram of food or stool specimen was weighed in a sterile container. For grinding the foods, the samples were transferred to a sterile mortar and 1 ml of cold gelatin diluent buffer was added. After homogenization, the samples were centrifuged at 12,000×*g* for 20 min, and the supernatant was tested by mouse bioassay for toxigenicity [6], [13]. Animal testing laboratory in this study was performed in accordance with the Institutional recommendations. The protocol was approved by the National Institute of Health Animal Care and Use Committee (NIH-ACUC, permit no. 55–009). After testing, the remaining laboratory animals were euthanized by CO_2_ gas administration according to the IACUC guidelines.

Half of the pellet from each sample was inoculated and grown on Egg Yolk Agar (EY) plates, Botulinum Selection Medium (BSM) (or Reinforced Clostridium Medium (RCM)) plates and incubated under anaerobic conditions at 35°C for two days. The remaining half of the pellet was divided in third and inoculated onto Chopped Meat-Glucose-Starch (CMGS) medium, CMGS medium heated at 80°C for 15 min (to select for *Clostridium* species), and Tryptone peptone glucose yeast extract trypsin (TPGYT). The inoculated samples were incubated under anaerobic conditions at 35°C for five days. Isolates were fully characterized by the methods of Dowell and Hawkins [11], and biochemical testing by Holdeman [12].

### Detection of *C. botulinum* Neurotoxins by Enzyme-linked Immunosorbent Assay (ELISA)

Samples of the 2010 outbreak were inoculated into Tryptone peptone glucose yeast extract trypsin (TPGYT) broth and the cultures were incubated for five to seven days at 35°C. The double sandwich ELISA tests [9] were provided by the Centers for Disease Control and Prevention (CDC), Atlanta. On each assay plate, there is positive control for botulinum neurotoxin type A, B, E, and F. The absorbance values ≥0.2 units were considered positive. The ELISA-positive samples were confirmed by neurotoxicity mouse bioassay.

### Mouse Bioassay

Typing and detection of BoNTs in serum and culture supernatants were performed by neurotoxicity mouse bioassay according to Hatheway et al. [6], [7]. The toxin type was identified by specific neutralization of biologic activity by using monovalent botulism antitoxin (available to qualified laboratories from the Biological Reagents Program, CDC). The concentration of monovalent reagents was approximately 10 IU/ml.

### DNA Extraction and Purification

Reference strains of *C. botulinum* (Table 1) were grown in Wilkins Chalgren broth (WB) [32], incubated anaerobically at 35°C for 48 hrs, and then harvested by low speed centrifugation. Pellets were resuspended in 1 ml of distilled water, and chromosomal DNA extracted using the QIAamp DNA Mini Kit (Qiagen Inc., Valencia, CA), according to the manufacturer’s instructions. DNA was eluted with 100 µl elution buffer and stored at 4°C until use.

**Table 1:** *C.botulinum* strains and sequences used in this study.

Bacterial strain	BoNT subtype	GenBankAccession No.	Origin (Country, Year)	Source or Reference	Testing
62A	A1	–	Stock strain	Osaka Prefecture,Japan	PCR, PFGE
Chiba H	A2	–	Honey (Japan, 1986)	Osaka Prefecture,Japan	
Renkon	A(B)^b^	–	Karahi renkon (Japan, 1984)	Osaka Prefecture,Japan	
Langeland	F	–	Duck liver paste (Denmark, 1958 )	Osaka Prefecture,Japan	
Osaka 05 nontoxic	Osaka 05nontoxic	–	Infant botulism (Japan, 2005)	Osaka Prefecture,Japan	
89E00061-2	B1	–	Infant botulism (United States,1989)	Osaka Prefecture,Japan	
Ginger	B2	–	Ginger (Japan)	Osaka Prefecture,Japan	
7H215S	B2	–	Honey (Japan)	Osaka Prefecture,Japan	
111 nontoxic	B2	–	Infant botulism (Japan, 1995)	Osaka Prefecture,Japan	
657	Ba	–	Unknown	Osaka Prefecture,Japan	
DMST27808	A	–	Unknown	NIH, Thailand	
DMST27809	B	–	Unknown	NIH, Thailand	
DMST 27810	F	–	Unknown	NIH, Thailand	
Osaka06	B2	AB302853	Infant botulism (Japan, 2006)	GenBank	Phylogenetic analysis
CDC1758	B1	EF033127	Unknown	GenBank	
Danish	B1	M81186	Unknown	GenBank	
Hall6517(B)	B1	EF028399	Unknown	GenBank	
CDC1656	B1	EF028396	Unknown	GenBank	
Prevot25 NCASE	B2	EF033129	Unknown	GenBank	
ATCC7949	B2	EF028395	Unknown	GenBank	
Smith L-590	B2	EF028398	Unknown	GenBank	
Prevot59	B2	EF033128	Unknown	GenBank	
CDC1828	B2	EF051571	Unknown	GenBank	
CDC6291	B2	EF028401	Unknown	GenBank	
Korean soil 1	B2	DQ417353	Korean soil	GenBank	
Korean soil 2	B2	DQ417354	Korean soil	GenBank	
CDC795	B3	EF028400	Unknown	GenBank	
CDC593	A(B)^b^	AF300466	Dog feces (United States, 1976)	GenBank	
CDC1436	Bivalent, AB^c^	AF295926	Stool sample (United States, 1977)	GenBank	
657Ba	Bivalent, Ba^c^	EF033130	Unknown	GenBank	
CDC588	Bivalent, Ab^c^	AF300465	Food borne (United States, 1976)	GenBank	
CDC3281	Bivalent, Bf^c^	Y13630	Infant botulism (United States, 1980)	GenBank	
ATCC17844	Nonproteolytic	EF028394	Unknown	GenBank	
Eklund17B (B257)	Nonproteolytic	EF051570	Unknown	GenBank	
10068	Nonproteolytic	EF028402	Unknown	GenBank	
Eklund17B	Nonproteolytic	X71343	Unknown	GenBank	
NTCT 2916	A(B)^b^	ZP 02612822	Unknown	GenBank	
Bac-04-07755	B7	JQ354985	Unknown	GenBank	
NCTC3807	B7	JN120760	Unknown	GenBank	
111	B2	AB302854,AB084152	Infant botulism (Japan, 1995)	GenBank	PCR, PFGE, Phylogenetic analysis
Okra	B1	AB232927	Food borne (unknown)	GenBank	
Osaka 05	Osaka 05	AB302852	Infant botulism (Japan, 2005)	GenBank	
Iwate 2007	A(B)^b^	AB665556	Stool sample	GenBank	
Saraburi S1, 2010	**A(B)** a	**JQ964804** a **, JQ964805** a	Foodborne (Thailand, 2010)	This study	
Maehongson S1, 2010	**B8** a	**JQ964806** a	Foodborne (Thailand, 2010)	This study	
Maehongson S1, 2010 nontoxic gene	**B8** a	**JQ964807** a	Foodborne (Thailand, 2010)	This study	

a The subtypes and GenBank accession numbers for strains determined in this study are indicated in boldface.

b *boNT*/A producing and unexpressed *boNT*/B gene possessing.

c Dual toxin producing strains; the major toxin type is indicated in uppercase letters and the minor type is indicated in lowercase letters.

### Identification of *C. botulinum* DNA by Multiplex PCR

The PCR was modified from the method of Lindström et al. [16] and used to identify BoNT types A through F. A 30 µl of reaction mix containing 0.5 µl of extracted DNA template, 0.1 µM of each primer, 200 nM of each deoxynucleotide triphosphate (dNTP), 5× Buffer, 1 U of Go*Taq*® DNA polymerase and 1.5 mM MgCl_2_ (Promega, Madison, WI, USA) was used. PCR was for 35 cycles, including a denaturation step at 95°C for 20 sec, annealing at 55°C for 30 sec and extension at 62°C for 2 min. The amplified PCR products were visualized by ethidium bromide in 2% agarose gels. All positive results were run through neurotoxicity testing for confirmation.

### Multiplex PCR for *boNT*/A and/B Subtyping

The method of Umeda et al. [33] was used for PCR typing of *boNT*/A, and Umeda et al. [25] for *boNT*/B typing except that 1 U of Go*Taq*® DNA polymerase and its associated buffer were used (Promega, Madison, WI, USA).

### Multiplex PCR of *ha33* and *p47genes* for Confirmation of *boNT/*A and *boNT*/B Typing

The PCR method was described by Umeda et al. [33] using 1 U of Go*Taq*® DNA polymerase and 1.5 mM MgCl_2_ (Promega, Madison, WI, USA). The amplified products of the *ha33* gene and *p47* gene confirmed as *C. botulinum* type A1 strain and type A2 strain, respectively. Both *ha33* and *p47* gene products indicated strain possessing both *boNT*/A and *boNT*/B genes (either expressed or unexpressed).

### Sequence Analysis of *boNT* and/or Nontoxic Component Genes for Strain Differentiation

Overlapping primer pairs covering the coding sequence of the different *boNT* and nontoxic component genes were designed for PCR amplification using sequence data available on GenBank (Table 1). Internal DNA oligomers were also designed within each amplicon for confirmatory sequence analysis in both directions. PCR was performed in a 50 µl reaction containing 1 ng of extracted DNA, 0.5 µM of each primer, 2.5 mM of MgCl_2_, 200 µM of each dNTP, 5× Buffer and 2.5 U of Go*Taq*® DNA polymerase and 1.5 mM of MgCl_2_ (Promega). Each PCR cycle consisted of denaturation at 94°C for 1 min, 55°C for 1 min, 72°C for 1 min and was repeated 30 times. Final extension was carried out 72°C for 10 min. Amplicons were directly sequenced by primer walking, and the sequence (in both directions) was confirmed using the ABI Prism BigDye Terminator Cycle Sequencing Kit (Applied Biosystems, Foster City, CA).

### Phylogenetic Analysis

DNA alignments were created with Clustal-W version 2.0 (http://www.clustal.org) [34]. A phylogenic tree was constructed based on the sequences of *boNT*/B (Table 1) using neighbor-joining method with 1,000 bootstrap replication, and the genetic distance matrix using Molecular Evolution genetic Analysis program (MEGA) version 5.0 [35].

### PFGE-probe Hybridization to Identify the Chromosomal or Plasmid Location of the *boNT* Gene

PFGE plug was prepared as described by Umeda et al. [25]. DNA was undigested and electrophoresed in a CHEF-DRIII apparatus (Bio-Rad Laboratories, Hercules, CA) through a 1% pulse field certified agarose gel (Bio-Rad Laboratories) in 0.5×Tris-borate-EDTA buffer (pH 8.3) at 14°C for and 6 V/cm. The switching times were ramped from 0.5 to 40 s for 18 hrs. The PFG was blotted onto Hybond N+ nylon membrane (Amersham Pharmacia Biotech, Little Chalfont, Buckinghamshire, UK) using 20× SSC for 18 hrs, and the DNA fixed to the membrane by UV irradiation. The *boNT*/A and *boNT*/B gene probes were prepared as reported by Takeshi et al. [36] and the *C. botulinum* Saraburi type A(B) and Maehongson B probes were prepared by digoxigenin (DIG)-labeling of the PCR products (Roche Diagnostics, Mannheim, Germany) as described by Umeda et al. [25]. Membranes were incubated at 55°C overnight with 20 ng/ml of each probe and then washed twice with 1× SSC containing 0.1% sodium dodecyl sulfate (SDS) for 5 min at room temperature, followed by two washes with 0.1× SSC/0.1% SDS at 55°C for 15 min. Membranes were rinsed in 100 mM malic acid, 100 mM Tris-HCl, 150 mM NaCl, pH 7.5 (Buffer 1), then blocked with 100 mM malic acid, 100 mM Tris-HCl, 150 mM NaCl, pH 7.5, 5% skim milk (Buffer 2) at room temperature for 30 min. Hybridization signals were detected with anti-DIG antibody (Roche Diagnostics, Mannheim, Germany) incubated for 30 min at room temperature in Buffer 2. Excess antibody was removed by washing twice with Buffer 1 plus 0.05% Tween-20 at room temperature for 10 min, followed by a wash in 1 M Tris-HCl, pH 9.5, 1 M NaCl (Buffer 3) at room temperature for 2 min. CDP star (Roche Diagnostics) was used at a 1∶1000 dilution in Buffer 3. The membrane was immersed for 2–3 min for signal development. The membrane was sealed in a plastic bag and exposed to X-ray film for 2–15 min.

### Nucleotide Sequence Accession Numbers

The nucleotide sequences determined in this work were submitted to the NCBI database with accession numbers JQ964804 to JQ964807 (Table 1).

## Results

### Isolation and Identification of *C. botulinum* and Detection of Neurotoxin Production

From Lampang province outbreak, *C.botulinum* type F was identified in one sample each of wild boar meat and sour pork by ELISA and mouse bioassay, but these samples were negative in the culture method. The positive result samples (one plastic-wrapped pork sausage (F1) and two stool specimens (S1, S2)) from Saraburi province outbreak and positive result samples (two fermented soy bean samples (F1, F2) and two stool specimens (S1, S2)) from Maehongson province outbreak were detected as *C. botulinum* type A and B by ELISA and mouse bioassay, and also isolated *C.botulinum* from positive samples (Table 2).

**Table 2:** Summary of botulism outbreaks in Thailand during 2010, by using culture, ELISA, mouse bioassays and molecular techniques.

Province/Outbreak perioid (2010)	No. of Cases	Sample	BONT type								Isolation *C.* *botulinum*	Molecular typing
			Mouse bioassay				ELISA					
			A	B	E	F	A	B	E	F		
Lampang 20April–17 May	7	Wild boar meat	D	D	D	A	−	−	−	+	−	−
		Sour pork	D	D	D	A	−	−	−	+	−	−
Saraburi 17–25 May	7	Plastic wrap pork sausage (F1)	A	D	D	D	+	−	−	−	+	A1(B)
		Stool specimen (S1)	A	D	D	D	+	−	−	−	+	A1(B)
		Stool specimen (S2)	A	D	D	D	+	−	−	−	+	A1(B)
Maehongson 16–18 December	5	Fermented soy bean sample (F1)	D	A	D	D	−	+	−	−	+	B8
		Fermented soy bean sample (F2)	D	A	D	D	−	+	−	−	+	B8
		Stool specimen (S1)	D	A	D	D	−	+	−	−	+	B8
		Stool specimen (S2)	D	A	D	D	−	+	−	−	+	B8

D = Dead, A = Alive+ = Positive, − = Negative.

### Identification of *C. botulinum* DNA, *boNT*/A and *boNT*/B Subtyping by Multiplex PCR

**Figure 1:**
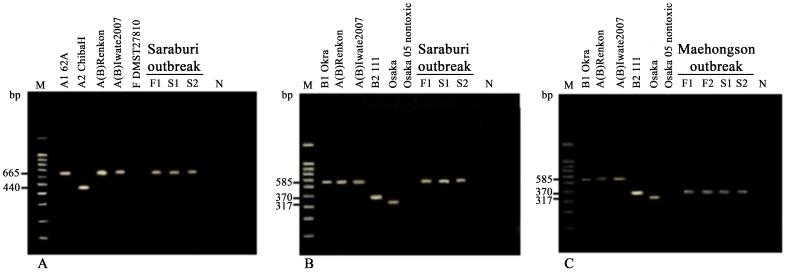
Multiplex PCR typing of *boNT*/A and *boNT*/B genes for Saraburi and Maehongson 2010 outbreak strains. The PCR pattern of Saraburi 2010 showed positive *boNT*/A1 amplicon (665 bp, gel A lanes 6–8) and *boNT*/B1 like amplicon (585 bp, gel B lanes 7–9). The PCR patterns of Maehongson 2010 indicated a *boNT*/B2-like gene was present as 370 bp amplicon (gel C, lanes 8–11).

PCR was used to identify *boNT* type, both the *boNT*/A and *boNT*/B genes from the Saraburi 2010 outbreak. BoNT type A was identified by ELISA and mouse bioassay but no BoNT B toxin was present (Table 2). Further molecular characterization of both *boNT* genes detected in the Saraburi 2010 strains indicated existence of types A1(B) (Fig. 1A and 1B). The Maehongson 2010 isolates were typed as B in the ELISA and mouse bioassay (Table 2) and the PCR results indicated a BoNT B2-like gene (Fig. 1C).

### Multiplex PCR of *ha33* and *p47genes* for Confirmation of *boNT/*A and *boNT*/B Typing

**Figure 2:**
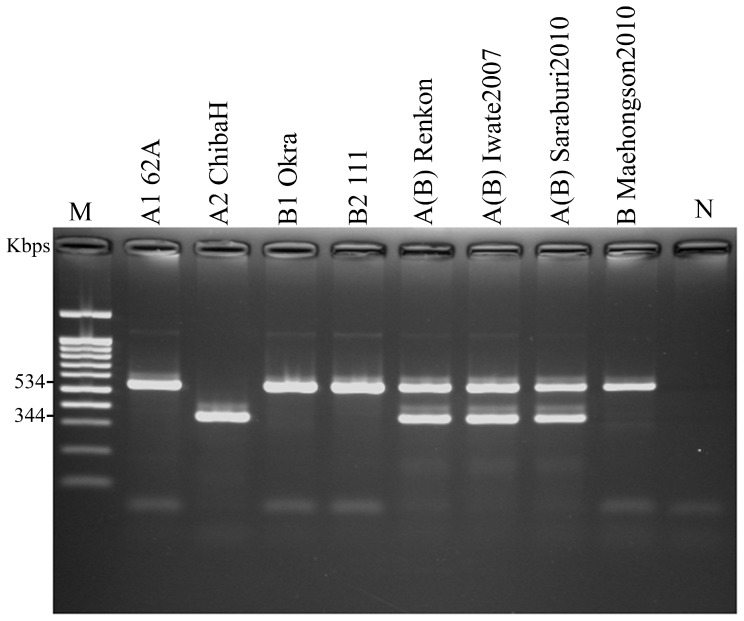
Multiplex PCR of *ha33* and *p47genes* for confirmation of *boNT/*A and *boNT*/B typing Isolate from Saraburi 2010 strain showed positive *ha*33 and *p47* amplicon (534 bp and 344 bp, lane7), *ha* 33 of *boNT*/A1 amplicon (534 bp, lane 1), *p47* of *boNT*/A2 amplicon (344 bp, lane 2).

Amplification of *ha*33 and *p*47 genes indicated presence of both *boNT*/A and *boNT*/B genes (either expressed or unexpressed) (Fig. 2). These results showed that toxin belonged to cluster type 3. Therefore, the Saraburi 2010 isolates are considered strain A1(B) [33].

### Sequence Analysis of *C. botulinum boNT*/A and *boNT*/B genes from Saraburi 2010 A1(B) and Maehongson 2010 (B) Strains

In the Saraburi outbreak the *C. botulinum* isolates were identified as type A1(B). The Saraburi 2010 *boNT*/A1 amino acid sequence showed 100% identity and similarity to *C. botulinum* type strain NTCT 2916 type A1(B), while the *boNT*/B amino acid sequence was 99.6% identical and showed 99.3% similarity to strain Iwate 2007, which was also an A1(B) type.

A BLAST analysis of *boNT*/B amino acid sequences from the Maehongson 2010 strain showed a 96% identity with other B2 strains. The Maehongson 2010 B strain had a 96% identity and 98.5% similarity to strain 111 (Table 3), the differences involved 52 amino acid residues (Table 4). Six residues were in the light chain (L) which was 441 amino acid long, and 46 residues in the heavy chain ((H), 850 amino acid). The N-terminus of the heavy chain (H_N_) (residues 442–861, a total of 420 amino acids) had 15 substitutions, and the C-terminus of the H-chain (H_C_) had 31/430 amino acid changes. Most of the changes in the H_C_, were concentrated in the H_CC_ subdomain (22/263 amino acid), while only 9/167 amino acid were different in the N terminal of the H_CN_. Comparing these domains to strain 111 and Maehongson 2010 showed amino acid identities of 98.60% (L), 95.12% (H), 97.13% (H_N_), 95.12% (H_CN_) and 93.04% (H_CC_) (Table 4).

**Table 3:** Percentage of identity and similarity of *boNT*/B gene and nontoxic component genes compared with strain Maehongson 2010 and Okra (subtype B1) and strain Maehongson 2010 and 111 (subtype B2).

	% Amino acid identity/similarity					
	HA cluster non- toxic genes (amino acid length)					Toxin gene (amino acid length)
Strains compared (type)	*ha*70(626)	*ha*17(146)	*ha*33(885)	*botR*(178)	*ntnh*(1197)	*boNT*/B(1291)
Maehongson 2010 (B) - 111 (B2)	98.1/99.2	97.3/97.1	84.6/90.5	96.6/98.4	98.4/99.2	96.0/98.5
Maehongson 2010 (B) - Okra (B1)	97.1/98.6	99.3/99.3	98.6/100	96.6/99.4	96.9/98.2	95.5/98.0

**Table 4:** Summary of *boNT*/B amino acid substitution between strain Maehongson 2010(MH 2010) and 111 in each domain (light chain and H_N_) or subdomain (H_CN_ and H_CC_ in H_C_). Hyphens indicate residues identical to those in strain 111.

Light chain (1–441)			H_N_ domain (442–861)			H_CN_ domain (862–1028)			H_CC_ domain (1029–1192)			H_CC_ domain (1193–1291)		
Position	MH 2010	111	position	MH 2010	111	position	MH 2010	111	position	MH 2010	111	position	MH 2010	111
147	G	E	476	N	D	919	V	F	1029	R	K	1200	H	Y
148	E	V	485	D	R	956	M	I	1032	R	G	1249	F	I
152	E	K	486	F	S	976	I	T	1072	E	K	1251	F	L
253	G	E	489	N	D	982	I	T	1105	S	N	1252	Q	K
389	D	N	496	D	N	993	K	E	1132	Q	N	1253	E	D
406	G	E	609	N	S	995	V	I	1138	D	N	1256	Y	N
			648	S	A	997	E	D	1148	K	R	1270	K	R
			750	S	N	1011	L	S	1174	L	S	1276	D	N
			768	I	V	1026	M	I	1176	Q	R	1291	T	I
			773	D	N				1188	Q	–			
			836	M	I				1189	Q	E			
			837	T	P				1190	K	E			
			851	K	E				1192	Q	K			
			852	M	I									
			853	V	F									

### Phylogenetic Analysis of *boNT/*B

**Figure 3:**
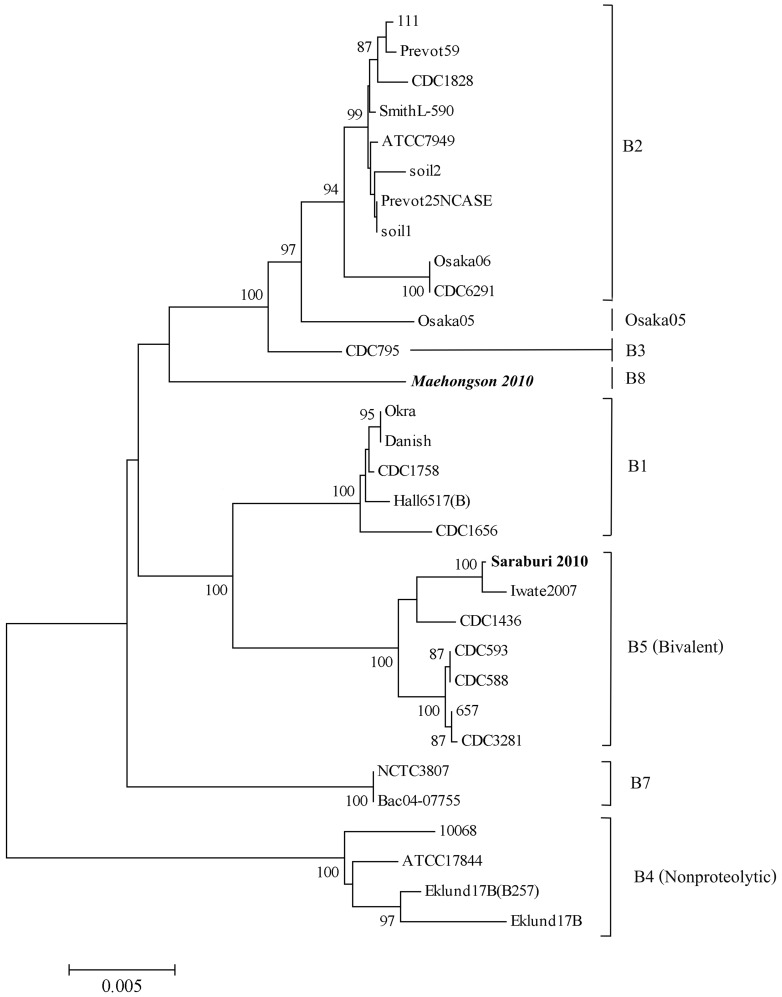
Phylogenetic analysis of *boNT*/B nucleotide sequences from various *C. botulinum* strains. The phylogenetic tree was generated using the neighbor-joining method in MEGA (v5) software. Bootstrap values (the percentage that each branch would occur after 1,000 bootstrap replicates) and genetic distance (bar) are shown. Clusters corresponding to different phylogenetic groups are labeled according to previous reports (Table 1). The toxin serotypes of the strains are shown on the right, and the Thai isolates from the 2010 outbreaks are highlighted.

A phylogenetic tree was constructed from the full length of nucleotide sequences of 31 *C. botulinum* type B strains (Fig. 3), including B1, B2, B3, nonproteolytic B4, bivalent B5, and B7 subtypes, along with Osaka 05, and Osaka 06 together with the type B isolates from the Thailand 2010 outbreaks (Table 1). The reliability of the tree topology was estimated by the bootstrap method using 1000 replicates. In this analysis, Saraburi 2010 was closest to Iwate 2007 isolate, clustering with the bivalent B5 strains. The Saraburi 2010 *boNT*/B showed a 99.3% similarity to Iwate 2007 at the nucleotide sequences level.

Strain Maehongson 2010 was slightly different from other B2 type strains as it did not cluster with them (Fig. 3). However, this isolate displayed a 98.5% similarity to the 111, a B2 strain. The greater than or equal to 4.0% difference in amino acid sequence and lack of clustering with known BoNT B subtypes has characterized the Maehongson strain as a unique BoNT B subtype (B8).

### Further Characterization of Maehongson 2010 Isolates by Comparison of Nontoxic Genes

Molecular characterization of the clusters of genes encoding the botulinum neurotoxin complex included the nontoxic components encoding the hemagglutinin genes (*ha*70, *ha*33, and *ha17*), the regulator gene (*botR*), and the nontoxic-non hemagglutinin gene (NTNH). The amino acid identities of the nontoxic components among Maehongson 2010, 111 (B2) and Okra (B1) are summarized in Table 3. The hemagglutinin cluster (*ha*70, *ha*17 and *ha*33) of strain Maehongson 2010 showed identities and similarities of 98.1%/99.2%; 97.3%/97.1%; 84.6%/90.5%, respectively with strain 111 (B2) at the amino acid level (Table 3). When compared to the Okra isolate, strain Maehongson 2010 showed amino acid identities and similarities of 97.1%/98.6%; 99.3%/99.3%; 98.6%/100%, respectively (Table 3). More in depth analysis showed that *ha*33 of Maehongson 2010 (B) had 47 amino acid substitutions compared to strain 111 (B2), but only four amino acid substitutions compared to strain Okra (B1). The gene *ha*17 of strain Maehongson 2010 had four amino acid substitutions compared to strain 111 and one amino acid substitution compared to strain Okra.

The amino acid sequence identities and similarities of *botR* and *ntnh* between Maehongson 2010 and the B2 111 and B1 Okra isolates are shown in Table 3.

### PFGE Probe Hybridization

**Figure 4:**
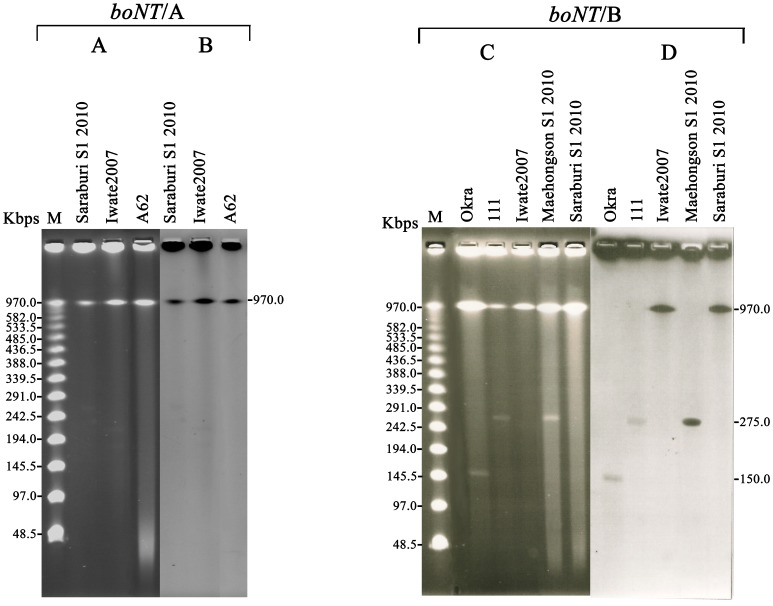
PFGE and Southern blot hybridization of undigested bacterial DNA from the different *C. botulinum* strains and outbreak isolates to identify *boNT* gene location. (A and C) undigested PFGE of isolated DNA (B) hybridization using a *boNT*/A probe (D) hybridization results with the *boNT*/B probe.

The PFGE and Southern blot hybridization against undigested DNA using *boNT*/A and *boNT*/B toxin-specific gene probes confirmed that the Saraburi 2010 *boNT*/A and *boNT*/B genes were located on chromosomal DNA as hybridization signal was detected at position 970 Kb (Fig. 4). In Maehongson 2010, *boNT*/B gene was located on a plasmid (275 Kbp, Fig. 4D) at the same position as in strain 111 (Fig. 4).

## Discussion

In this study, we examined the *C. botulinum* isolates from three different foodborne outbreaks in Thailand during 2010. The first outbreak occurred at Lampang province in April 2010, and was caused by *C. botulinum* type F which was typed by ELISA and mouse bioassay. The second and third outbreaks occurred in Saraburi and Maehongson provinces during May and December 2010, respectively. The *C. botulinum* isolates from Saraburi were identified as type A by mouse bioassay and ELISA, and the PCR patterns were consistent with type A1(B). The Maehongson isolates were determined as type B using the PCR assays for toxin and nontoxic gene cluster components.

The typing method of Umeda et al. [25], [33] has several limitations in differentiating the *boNT*/A1, A2 and *boNT/*B1 from B2 or B6 types. The typing for *boNT*/A3–A5, B3–B5 or B7 has not been published. Moreover, the primers for typing *boNT*/B1 could amplify the sequence position from 3256 to 3840 of strains Eklund17B (B4), ATCC 17844 (B4), Iwate 2007(B5), 657Ba(B5), CDC 593(B5) and CDC 1436(B5) which were almost the same sequences similar to that of strain Okra (B1). Studies of Franciosa et al. [37] and Cordoba et al. [38] confirmed the presence of silent or unexpressed *boNT*/B gene in some *C. botulinum* type A strains using PCR-restriction fragment length polymorphism and DNA probs. In this present finding, we have also shown that the Saraburi 2010 isolates carried a silent toxin encoding genes (Fig. 2), which were designated as toxin cluster type 3 (the *ha*33+and *p*47+ genes). In addition, Franciosa et al. [38] found that two strains of *C.botulinum* type A (strains CDC-1882 from infant botulinum and CDC-1903 from food borne botulism) were negative for *ha*33 and positive for *p*47 genes (like type A2 strains), and displayed a *boNT*/A1 PCR-RFLP type. This result was remarkable since this gene combination has been reported only for the *boNT*/A gene cluster of *C.botulinum* type A(B) and Ab strains. Franciosa et al. [38] hypothesized that a third *boNT*/A gene cluster may exist among strains of *C.botulinum* type A which were confirmed as toxin cluster type 3. Sequencing of the *boNT*/A and *boNT*/B genes from Saraburi 2010 revealed the complete identity at the amino acid level and similarity of the *boNT*/A1 gene to the Iwate 2007 and NTCT 2916 strains (results not shown), while 99.6% identity and similarity of the *boNT*/B at the amino acid level were observed with Saraburi 2010 and Iwate 2007 strains. Thus, the silent B gene in Saraburi 2010 strain was more closely related to BoNT B5 (bivalent) which was shown in the phylogenetic tree (Fig. 3). *C.botulinum* strains that contain the genes of two toxin types probably evolve when one toxin type strain acquires the genes encoding different antigenic types. This transfer possibly involved not only the neurotoxin gene, but genes of the entire cluster that could differ in their arrangement and content. The mechanism of transfer is not known but would require a vector such as bacteriophages or conjugative transposable elements that are capable of transferring large regions of donor DNA [39]. Hutson et al. [40] found that the most striking difference in isolates with silent neurotoxin gene sequences was the presence of a stop codon and sequence deletions in the gene of the A(B). These types of changes could affect transcription initiation or termination, or might produce a truncated protein with decreased toxicity. Alternatively, the quantities of toxin molecule synthesized might be similar, but their specific toxicities were different due to the changes in protein structure or post-translational modification. *C. botulinum* strains, the influences of amino acid substitutions/deletions on biological activity of the toxin molecule are not known.

The chromosomal or plasmid location of each *boNT* gene was examined to determine the similarity if any [41]. The location of the *boNT* gene and its associated nontoxic component genes varied among serotypes and strains. The *boNT*/A1, *boNT*/A1(B), *boNT*/A2 and *boNT*/F gene types are located in the bacterial chromosome, while the *boNT*/A3, *boNT*/A4, *boNT*/bvB, *boNT*/B1, *boNT*/Bf genes are located on plasmids [42]–[45]. The *boNT*/np B and *boNT*/G genes were identified in plasmids [46], [47], while the *boNT*/C and *boNT*/D genes were carried on bacteriophage [48], [49]. The Southern blot analysis from the undigested PFGE showed that both the *boNT/*A and *boNT*/B genes of Saraburi 2010 isolate were chromosomally located (Fig. 4B and 4D).

There are currently seven known subtypes within the *boNT*/B serotype, B1–B7, and they exhibit 1.5–7% sequence variations at the amino acid level. The amino acid variation within a subtype is much less (1.5%) in B2 and around 1.7% with other subtypes. The amino acid identity and similarity between the *boNT*/B of strain Maehongson 2010 and strain 111 were 96% and 98.5%, respectively. These amino acid differences indicate that Maehongson 2010 is a new BoNT B subtype or toxin variant [24], [50]. The amino acid substitutions in strain Maehongson 2010 were assembled in the heavy chain (Table 4). This is supported by the phylogenetic analysis that places the strain Maehongson 2010 in a different cluster to other B2 subtypes (Fig. 3). The origin and mechanism of acquisition of the neurotoxin gene cluster are intriguing. The gene arrangement and sequence homologies raise interesting questions. It appears likely that acquisition of the neurotoxin cluster occurred as a series of evolutionary events more recently than the divergence of the neurotoxigenic clostridia from a common precursor organism, as the neurotoxin gene clusters show relatively high homology. However, the origin for these genes are unknown [41], [51]. On the other hand, the sequence differences between *boNT*/B1 and *boNT*/B2 genes indicate differences in molecular size, antigenicity and possibly receptor recognition at the neuromuscular junction [41]. The potency of BoNT depends on its enzymatic activity and high affinity binding to neurons.

BoNTs are synthesized as single chain peptides with molecular mass of ∼150 KDa that are proteolytically activated into a light chain (L; 50 KDa) and a heavy chain (H; 100 KDa) and linked by a disulfide bond. Functional activities are attributed to certain domains of BoNT. The H chain serves to transfer and deliver the L-chain into the cytosol of neuronal cells [52], the amino-terminal (H_N_) is associated with translocation of the L chain from the lumen of an acidic intracellular compartment into the cytosol subsequent to cell binding and receptor-mediated endocytosis. The carboxyl-terminal domain of the H-chain (H_c_) displays highly selective binding for neurons, particularly those of the cholinergic system [53]. Therefore, the high amino acid variability presented in the H_cc_ terminal (22/263 residues) of strain Maehongson 2010 could affect neuronal binding and antibody specificity of the neurotoxin. This information may be important for the identification of more specific and efficient immunoglobulin than currently used equine immunoglobulin. Various monoclonal antibodies have been proposed as immunoglobulin treatments for botulism [54]–[56]. If there is any differential binding of these antibodies to the different subtypes, selection of antibodies for treatment should be accurate, as they may not be effective for neutralizing all subtypes of BoNT within a serotype [57].

BoNTs are associated with non-toxic proteins (ANTPs) that form complexes of various sizes that facilitate the translocation of the toxin [58]. Genomic analyses have provided evidence of horizontal gene transfer, site-specific insertion, and recombination events, which contribute to the variations among the neurotoxins, the toxin gene clusters and the bacteria in which they occurred [39]. The *boNT* genes lie in the 3′ portion of the locus and are immediately preceded by the non-toxic and non-hemagglutinin gene components (NTNH). The *ntnh* and *boNT* genes are transcribed in the same orientation, and the genes encoding hemagglutinin components (HA33, HA17 and HA70 in *C. botulinum* A) are upstream of these and are transcribed in the opposite direction. Our results showed high amino acid level identity and similarity of the *ha*33 and *ha*17 genes (98.64% and 99.32% identities, respectively) between B (Maehongson 2010) and B1 (Okra), but the *ha*70, *botR* and *ntnh* genes of B (Maehongson 2010) showed greater similarity at the amino acid level to B2 (111) (Table 3), reinforcing the idea that strain Maehongson 2010 could be different from others type B2. The variation in the composition of the hemagglutinins in the BoNT complexes might affect both the functionality and potency of neurotoxin itself [59], and there is an indication that the hemagglutinins may also facilitate the absorption of BoNT from intestines into the bloodstream [58].

The *boNT* genes are located within plasmids of varying sizes (47.6–270 Kb) [46], [60] or within the bacterial chromosome. The probe for the BoNT B genes for strains 111 and Maehongson 2010 hybridized to a band of ∼275 Kbp in the PFGE probe experiment (Fig. 4D). This size is similar to that of plasmids containing *boNT/*A3, Ba, and Bf genes (∼245–270 Kbp) [39], [46]. No chromosomal band was hybridized to the *boNT*/B probe, indicating that the plasmids are not integrated into the chromosome. The plasmid may play an important role in mediating genetic transfer within and among the bacterial genomes [61], and this has important implications in the evolution and pathogenicity of *C. botulinum*, including acquisition and expression of the toxin [62]. A future challenge has to consider whether the *boNT*-encoding plasmids play a role in the development and/or flexibility of *C. botulinum,* potentially increasing its adaptability to certain niches, such as food matrixes and specific environments, and ultimately contributing to the disease of botulism. Molecular genotyping of *C. botulinum* is an important tool contributing to our general understanding of the organism and the pathological consequences of infection. It is likely that differences in toxins will require a full understanding of specific antibody therapeutics.
